# RecX Facilitates Homologous Recombination by Modulating RecA Activities

**DOI:** 10.1371/journal.pgen.1003126

**Published:** 2012-12-20

**Authors:** Paula P. Cárdenas, Begoña Carrasco, Clarisse Defeu Soufo, Carolina E. César, Katharina Herr, Miriam Kaufenstein, Peter L. Graumann, Juan C. Alonso

**Affiliations:** 1 Department of Microbial Biotechnology, Centro Nacional de Biotechnología, Consejo Superior de Investigaciones Científicas, Madrid, Spain; 2 Mikrobiologie, Fakultät für Biologie, Universität Freiburg, Freiburg, Germany; Agency for Science, Technology, and Research, Singapore

## Abstract

The *Bacillus subtilis recH*342 strain, which decreases interspecies recombination without significantly affecting the frequency of transformation with homogamic DNA, carried a point mutation in the putative *recX* (*yfhG*) gene, and the mutation was renamed as *recX*342. We show that RecX (264 residues long), which shares partial identity with the Proteobacterial RecX (<180 residues), is a genuine recombination protein, and its primary function is to modulate the SOS response and to facilitate RecA-mediated recombinational repair and genetic recombination. RecX-YFP formed discrete foci on the nucleoid, which were coincident in time with RecF, in response to DNA damage, and on the poles and/or the nucleoid upon stochastic induction of programmed natural competence. When DNA was damaged, the RecX foci co-localized with RecA threads that persisted for a longer time in the *recX* context. The absence of RecX severely impaired natural transformation both with plasmid and chromosomal DNA. We show that RecX suppresses the negative effect exerted by RecA during plasmid transformation, prevents RecA mis-sensing of single-stranded DNA tracts, and modulates DNA strand exchange. RecX, by modulating the “length or packing” of a RecA filament, facilitates the initiation of recombination and increases recombination across species.

## Author Summary

This study describes mechanisms employed by the bacterium *Bacillus subtilis* to survive DNA damages by recombinational repair (RR) and to provide genetic variation via genetic recombination (GR). At the center of homologous recombination (HR) is the recombinase RecA, which forms RecA·ssDNA filaments to mediate SOS induction and to promote DNA strand exchange, a step needed for both RR and GR. Genetic data presented here highlight the complexity of the network of RecA accessory factors that regulate HR activities, with RecX counteracting the role of RecF in SOS induction. The absence of both RecA modulators, however, blocked RR and GR. Insights into the spatio-temporal recruitment of RecA to preserve genome integrity, to overcome the barriers of gene flow, and its regulation by mediators and modulators are provided. Chromosomal transformation, which declines with increasing evolutionary distance, depends on HR. Indeed, the presence of the RecX modulator decreases the genetic barrier between closely related organisms. The role of RecA mediators and modulators on the preservation of genome integrity and long-term genome evolution is discussed.

## Introduction

The bacterial RecA recombinase (homologue to human RAD51 and DMC1), arranged as higher-order oligomers assembled on tracts of single-stranded (ss) DNA, is involved in the DNA strand exchange reaction to warrant genome integrity by recombinational repair (RR), and genetic diversity by genetic recombination (GR). Template-dependent RR preserves the integrity of the genetic information, re-establishes replication and ensures proper chromosomal segregation. In contrast, GR, which occurs in species that can exchange chromosomal DNA segments, is an important mechanism for natural variation among prokaryotes and plays an important role in the dissemination of important traits, such as antibiotic resistance, virulence determinants and metabolic pathways involved in adapting to environmental niches. There are three modes by which bacteria can exchange chromosomal DNA segments: viral-mediated transduction, which may be limited by the viral host range and by the host-encoded restriction system, conjugation and natural transformation. *Bacillus subtilis* transformation or *Escherichia coli* conjugation catalyze unidirectional integration of chromosomal ssDNA at a frequency that decreases exponentially with the increasing degree of DNA sequence divergence between donor and recipient reviewed in [1], [2]. In *E. coli* the extent of genetic isolation by HFR conjugation is determined by the activity of the mismatch repair system, and requires DNA replication and recombination functions (specifically requires overproduction of the RecA protein) [3], [4], [5]. *B. subtilis* natural transformation, which can take DNA of any source, is insensitive to restriction endonucleases and to mismatch repair functions, and shows no obvious requirement for extended DNA replication [2], [6]. RecA-dependent homologous recombination (HR) rather than mismatch repair seems to control the extent of genetic isolation during natural transformation [6]. Here, a specific set of recombination functions, some of which are induced by natural competence (e.g., SsbA, SsbB, DprA [Smf or CilB], RecA, CoiA), are mainly located at the cell poles (namely SsbB, DprA, RecA, CoiA and RecU) where the DNA uptake machinery is located [7]–[11]. Except for *recA* and *dprA* mutations, the *B. subtilis* chromosomal transformantion frequency with homogamic DNA in single *rec*-deficient strains, classified within the α (*recF*15 or Δ*recO*), β (*addA*5), γ (*recH*342), δ (Δ*recN*), ε (Δ*ruvAB*, Δ*recU*), ζ (Δ*recQ*) or η (Δ*recG*) epistatic groups, does not vary more than 3-fold relative to the *rec* ^+^ value [12]–[15]. The absence of RecA blocks chromosomal transformation, and the absence of DprA results in a 50-fold reduction relative to the *rec* ^+^ value [10], [16]–[18]. From those *rec*-deficient strains tested, the frequency of interspecies gene exchange deviated significantly from the *rec* ^+^ strain only in the *recH*342 mutant strains [6]. The frequency of transformation with divergent donor DNA decreased >20-fold in the *recH*342 strain relative to the *rec* ^+^ value, without affecting the frequency of transformation by closely related donors [6], suggesting that HR introduces barriers to genetic exchange, and that the “RecH342” mutation contributes to sexual isolation. Very little is known about the mutation(s) present in the *recH*342 (BG119) strain, but the phenotype(s) associated with it suggested that the function(s) affected in this strain might act as an accessory factor by regulating the formation of an active RecA filament [19].

Why Does RecA need accessory factors? The essential single stranded binding (SSB) protein (termed SSB in *E. coli*, SsbA in *B. subtilis* or RPA in eukaryotes), which is ubiquitous in all living organisms, is involved in multiple pathways of DNA metabolism, including DNA replication, RR and GR reviewed in [20]. The majority of naturally competent bacteria encode a second non-essential protein, termed SsbB [21]. Biochemical studies have shown that the SSBs proteins, which bind to ssDNA and remove secondary structures, limit RecA loading onto ssDNA, as a consequence of the higher affinity and faster binding kinetics, so that the net result is a SSB-coated ssDNA reviewed in [20]–[24]. Furthermore, RecA·ssDNA filament elongation is blocked by DNA secondary structures, whereas assembly of SSB proteins is not, and SSB proteins contribute to the removal of secondary structures upon ssDNA binding, hence the RecA·ssDNA filaments formed on SSB-coated ssDNA after removal of the SSB protein(s) are more efficient than those formed by RecA alone reviewed in [20]–[24]. To overcome the effect of a SSB protein on RecA nucleation onto ssDNA, and RecA filament formation, a series of RecA accessory factors regulate such stage reviewed in [22]–[25]. These factors can be divided into two broad classes: those that act before RecA nucleation by promoting assembly of RecA onto SSB-coated ssDNA (termed RecA mediators), and those that act after RecA nucleation and during homology search and strand exchange, by promoting RecA·ssDNA filament assembly and disassembly (termed RecA modulators) [25]. Genetic recombination and RR share some accessory factors, but others are specific for each event. The most ubiquitous RecA mediators are RecO and RecR, which are involved both in RR and GR. The role of RecBCD (counterpart of *B. subtilis* AddAB) and RecF as RecA mediators is less conserved and less well-understood in bacteria other than γ-Proteobacteria [26]–[28]. DprA is an ubiquitous RecA mediator that plays a relevant role during GR (see above). The RecA modulators RecF and RecX are widely present in bacteria, but very little is known about their *in vivo* role [9], [19], [22]. *In vitro* RecX*_Eco_* destabilizes the RecA*_Eco_*·ssDNA filaments and RecF*_Eco_* antagonizes this effect [29], but RecA*_Eco_* foci formation (nucleation onto ssDNA?) decreases in the Δ*recX_Eco_*, but increases in the *recF*4115*_Eco_* context [30]. The difference between the simplified *in vitro* system and *in vivo* could be related to the presence of other RecA modulators in the γ-Proteobacteria, as DinI*_Eco_* and RdgC*_Eco_*, whose presence in bacteria of the Firmicutes Phylum is not obvious reviewed in [22].

In *B. subtilis*, cytological studies have shown that RecN, RecO, RecR, RecA and RecF form a discrete focus on the nucleoid in response to DNA damage. By observing the localization and temporal order of recruitment, we learned that these proteins co-localize to a defined DNA double-strand break (DSB), with RecN localizing first, while RecO, RecR and RecA localize later, followed by RecF [31], [32, our unpublished results]. Concomitantly with RecF assembly, the RecA foci are converted onto highly dynamic filamentous structures (termed threads) across the nucleoid that are disassembled 120 min later [32]. Biochemical studies suggested that a dynamic RecA·ssDNA filament with an “effectual length” is essential for SOS induction, template-dependent RR and for programmed GR [19], [24]. Previously, it has been shown that: i) a subset of RecA functions shows optimal activity at a high ssDNA/protein ratio, which might pack less RecA per unit length of ssDNA, and requires NTP hydrolysis, whereas other catalytic activities are optimal in RecA-saturated complexes that require NTP, but do not hydrolyze it [33], and ii) RecA-mediated SOS induction requires an extended filament conformation, but no ATP hydrolysis [34], [35]. For the SOS induction an extended and saturated RecA·ssDNA filament [33], [35], is essential for LexA repressor self-cleavage [36]. The absence of LexA increases the expression of SOS genes reviewed in [37]. In *E. coli*, RecX inhibits the RecA coprotease activity of RecA *in vitro* and *in vivo*, but a null *recX* mutant (Δ*recX*) strain shows no obvious phenotype [38], [39].

In *E. coli* and *B. subtilis* the SOS response is reduced and delayed in the absence of RecF, RecO and RecR [40], suggesting that these products could work as mediators and/or modulators. This is consistent with the observation that certain RecA mutant proteins act as suppressors of the *recO*, *recR* or *recF* defect [41], [42]. These RecA mutant variants showed the unassisted ability to displace the SSB protein [43], suggesting that specialized RecA mediators and/or modulators that regulate RecA activities are necessary to avoid the potential hazard that could be caused by miss-regulation of HR [22].

Biochemical studies with protein of *E. coli* origin, have shown that RecO, alone or in concert with RecR, aids RecA to overcome the limitation imposed by the SSB protein, and loads RecA onto the ssDNA [26], [44]–[47]. Then, RecX inhibits the strand exchange reaction by blocking RecA·ssDNA filament formation or facilitating RecA filament disassembly [38], [39], [48], [49], whereas RecF, which physically interacts with RecX, actively participates in the addition of RecA monomers to the nucleoprotein filament, by inhibiting the effect of RecX [29]. These proteins might also modulate the RecA/ssDNA ratios (packing) or the length of the RecA·ssDNA filament (see above).

During programmed GR in *B. subtilis* competent cells, the internalized ssDNA should be coated by one of the SSB proteins (SsbA or SsbB). RecO, alone or in concert with RecR, or DprA aids RecA to overcome the limitation imposed by SsbA or SsbB (or both in concert) and loads RecA onto ssDNA tracts [18], [50]. Then, RecA polymerizes on the filament (RecA threads?) and rapidly scans for a homologous dsDNA segment in the recipient that eventually binds to RecA to allow for strand exchange. Here, one strand of the recipient duplex unbinds from its partner and pairs with the internalized ssDNA. Note that henceforward in this paper, and unless stated otherwise, the indicated genes and products are of *B. subtilis* origin.

To gain insight into the initial state of RecA regulation, we have *in vivo* characterized the function(s) impaired in the *B. subtilis recH*342 strain. We have identified the mutation of the *recH*342 strain, which maps in the putative *recX* (*yfhG*) gene, so that the mutation was renamed as *recX*342. We have deleted the putative *recX* gene (Δ*recX*), and investigated the *in vivo* role of RecX to gain insight in the regulation of RecA activity by analyzing its effect in induction of the SOS response, RR and GR. Our work reveals that the absence of RecX reduces the threshold for damage-dependent SOS response, the *recF*15 mutation delays it, and the effect observed in the single mutants is overcome in the Δ*recX recF*15 context. We show that RecA·ssDNA filament necessary for SOS induction is not sufficient for RecA-mediated strand exchange. Here, RecX might act by increasing the stability of the joint molecule or by affecting the length of the minimum efficiently processed segment (MEPS) and indirectly removes a barrier for genetic exchange. We propose that RecA exerts a negative effect on plasmid transformation and RecX suppresses it. Our work demonstrates that RecX facilitates HR by modulating RecA activities and plasmid establishment by inhibiting RecA.

## Results

### The *recH*342 mutation maps in the putative *recX* gene

The radiation-sensitive *rec*342 mutant strain, which was isolated in late sixties [51], bears two separable mutations. One mutation, which leads to methyl methanesulfonate (MMS) sensitivity, was termed *recH*342 (BG119 strain) and classified within the γ epistatic group [12], . To identify the mutation(s) present in the *recH*342 strain (BG119) nucleotide sequence analysis and whole-genome comparisons (Genome Analyzer, Illumina) were performed in parallel with the isogenic *rec* ^+^ (Reference strain [BG214]). The isogenic BG119 (*recH*342) showed 9 differences with the BG214 (*rec* ^+^) strain, resulting in 5 amino acid changes, 3 intergenic mutations and 1 silent mutation (Table 1). The DNA repair phenotype observed in *recH*342 strain could be attributed to the substitution of Leu for Pro (L101P), in a conserved region of the YfhG protein (see Figure S1). YfhG shares a low but significant level of identity with genuine RecX proteins [53] (see below). The BG119 strain also carried a point mutation (P236S) in a variable region of the DNA translocase SftA (Table 1). SftA, which is required for coupling chromosomal segregation and cell division, assists the tyrosine recombinases in the resolution of chromosomal dimers reviewed in [54]. Since the mutations present in *recH*342 did not confer a significant chromosomal segregation defect [55], [56], and a plasmid-borne *sftA* gene failed to complement the MMS-sensitive phenotype of *recH*342 cells (data not shown), we assumed that the mutation in the *sftA* gene should not be responsible for the observed phenotype.

**Table 1:** Overview of the mutations present in the *recH*342 strain identified by nucleotide sequencing.

Genome position^a^	Locus^a^	Gene	Gene product	Reference^b^	Test^b^	Amino acid change^c^
330380	329774–330739	*ldh*	L-lactate dehydrogenase	AAA (607)	GAA	K203E
925934	925633–926427	*yfhG*	Hypothetical recombination regulator, RecX	CTT (302)	CCT	L101P
1041854	1041994–1042842	*dat*	Intergenic, D-alanine aminotransferase	T	G	-
1283580	1283463–1284362	*yjfC*	Hypothetical protein BSU12130	ACC	GCC	T40A
2893906	c2894709–2893681	*ilvC*	Ketol-acid reductoisomerase	GCG (207)	GCA	A69A (silent)
2982417	c2982269–2981151	*citZ*	Intergenic, citrate synthase	T	A	-
2982437	c2983067–2982603	*ytwI*	Intergenic, hypothetical protein	T	C	-
3024588	c3025266–3023677	*ytcI*	Acyl-CoA synthetase	GTA (685)	ATA	V227I
3051461	c3052583–3049725	*sftA*	DNA translocase	CCA (1123)	TCA	P236S

a The single genome position presenting the mutated nucleotide position, and the locus interval are shown, where the c denotes the complementary strand.

b The underlined bases represent the bases that were present in the Reference (BG214) and the Test (*recH*342) sample, and between parentheses the exchanged nucleotide number.

c The position and substitution of the mutated residue.

**Figure 1:**
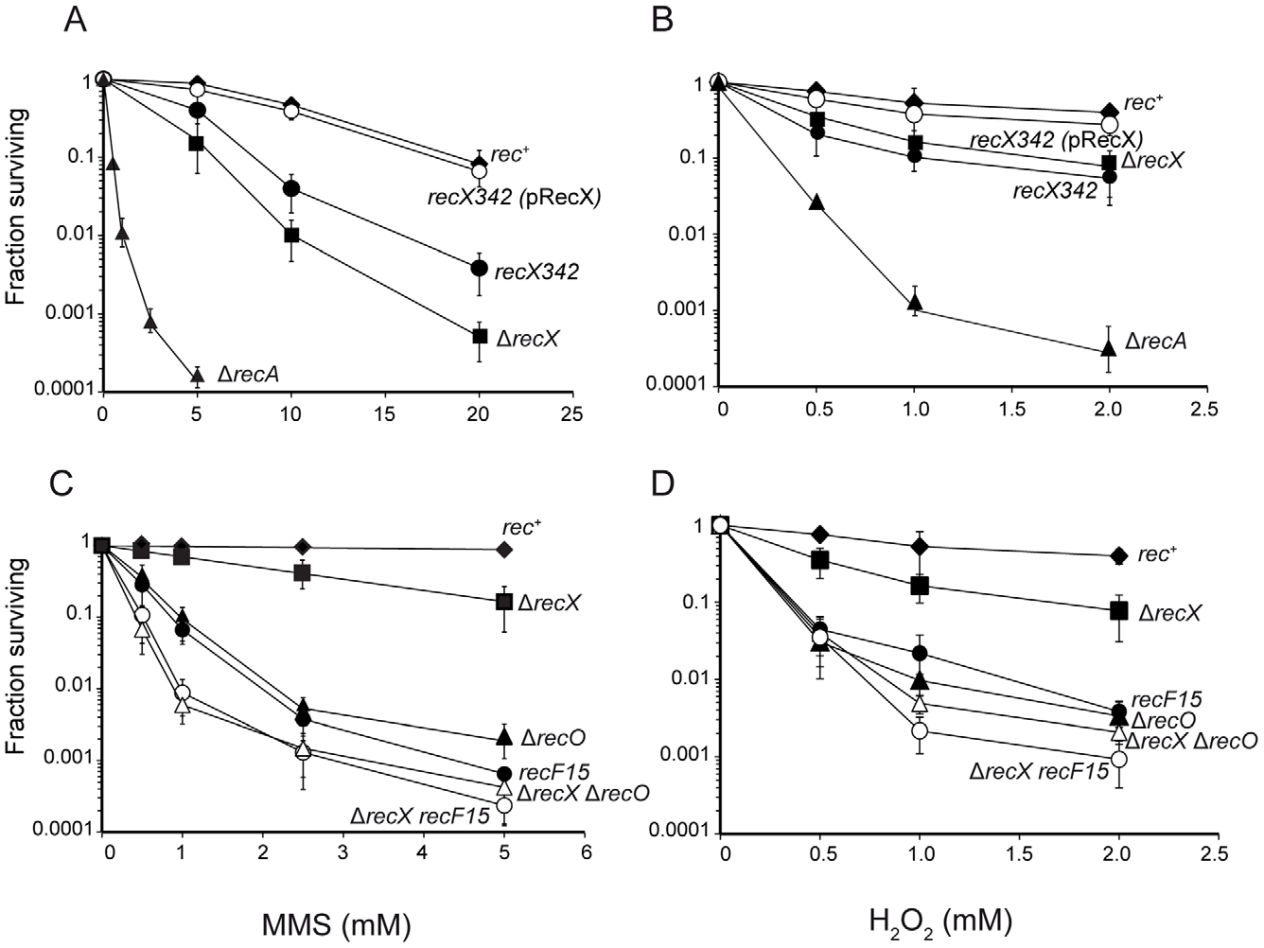
Survival curves of *B. subtilis* cells after exposure to an acute dose of MMS or H_2_O_2_. Cells were grown to OD_560_ = 0.4 in LB medium and exposed to increasing concentrations of MMS (A and C) or H_2_O_2_ (B and D) for 15 min. The strains used are indicated by the relevant mutant phenotype. The *recX*342 (pRecX) strain bears a plasmid-borne *recX* gene. The results are the average of at least five independent experiments and the standard errors are indicated.

To test whether putative RecX is involved in RR and/or GR and if it complements the *recH*342 defect, a null *recX* (Δ*recX*) mutant strain was created, and a plasmid-borne *recX* gene was introduced into the *recH*342 context. As revealed in Figure 1A, the Δ*recX* or *recH*342 mutation rendered cells sensitive to MMS and H_2_O_2_ when compared with *rec* ^+^ cells. A plasmid-borne *recX* gene in *recH*342 cells restored *rec* ^+^ levels of MMS or H_2_O_2_ resistance (Figure 1A), thus the *recH*342 mutation was renamed as *recX*342. This is consistent with the observation that the physical mapping of *recX* gene, at 79° [57], is in good agreement with the genetic map of the *recH*342 mutation, in the *tre* - *glyB* region (82° interval) by PBS1 transduction [58].

To ascertain the role of RecX in RR, we transferred the Δ*recX* mutation into strains lacking RecA accessory proteins (e.g., *recO*, *recF*) and assayed the ability of these strains to resist the acute exposure to MMS or H_2_O_2_. The Δ*recA* strain was used as control. Upon exposure to varying concentrations of DNA damaging agents, the Δ*recX* strain was moderately sensitive to both drugs when compared with the very sensitive *recF*15 or Δ*recO* strains or the extremely sensitive Δ*recA* strains (Figure 1A and 1B). The absence of both RecX and RecO or RecX and RecF, increased the sensitivity of the double mutant strains (Figure 1B) to the levels of Δ*recA* (Figure 1A). It is likely that Δ*recX* is not epistatic with Δ*recO* or *recF*15 (classified within the α group), and that the three functions are essential for RecA-mediated DNA strand exchange.

Genes others than *recA*, which are exclusively involved in HR have been placed into seven different epistatic groups. Except *recX*342 (epistatic group γ) and *recF* and *recO* (α group) described above, the different epistatic groups and the genes included within them are: *addA* and *addB* (β); *recN* (δ); *ruvA*, *ruvB* and *recU* (ε); *recJ*, *recQ* and *recS* (ζ) and *recG* (η) (Figure S2) [19]. The Δ*recX* mutation was moved into a representative of each epistatic group [Δ*addAB* (β), Δ*recN* (δ), Δ*recU* (ε), Δ*recJ* (ζ) or Δ*recG* (η) epistatic group] (C.E.C, G. Garaulet, C. Marchisone and J.C.A., unpublished results). The single and double Δ*recX* mutant strains were assayed to resist the acute exposure to MMS and H_2_O_2_ or the chronic exposure to mitomycin C (MMC) and H_2_O_2_. As previously shown for the *recX*342 mutation [52], [56], [59], Δ*recX* was neither epistatic with Δ*addAB* (β), Δ*recN* (δ), Δ*recU* (ε), Δ*recJ* (ζ) nor with Δ*recG* (η epistatic group) (C.E.C., G. Garaulet, C. Marchisone and J.C.A., unpublished results).

### Firmicutes *recX* gene is distantly related to Proteobacterial *recX*

The *recX* gene shows a high ubiquity among Bacteria [53]. It is predicted to be missing only in bacteria of the Cyanobacteria and Chlamydiae Phyla and in some Classes of the Proteobacteria (e.g., α-Proteobacteria), Firmicutes (e.g., Mollicutes) or Spirochetes Phyla. The RecX orthologues (197 orthologues analyzed) showed only a limited degree of identity among Phyla, but a high degree of identity was observed between the different Classes of the same Phylum [53], [60]–[62], suggesting a high divergence or more than one possible evolutionary pathway.

The RecX orthologues were classified using a length bias criterium (Figure S1). With few exceptions (e.g., RecX of the *Yersinia* Genera that are significantly longer, >180-residue long polypeptide), RecX of the Proteobacteria Phylum (87 orthologues analyzed) are relatively small proteins (<170-residue long polypeptides), and share a significant degree of identity (>25%) among them [53], [62] (Figure S1). The structure of RecX*_Eco_* revealed that it is a modular protein consisting of three tandem repeats of a three-helix motif (R1α1-3, R2α1-3 and R3α1-3) (Figure S1) [63]. These RecX proteins can be further divided in two subgroups represented by *E. coli* (*Eco*) and *N. gonorrhoeae* RecX (*Ngo*) (Figure S1). The *recX* gene of the former group is located immediately downstream of *recA*, forming a single transcriptional unit as in *E. coli*. RecX of the latter group, which is not part of the SOS response, is located elsewhere in the genome, as in *Neisseria ssp*. [60], [61].

RecX of the Actinobacteria Phylum (15 orthologues analyzed), represented by *Mycobacterium tuberculosis* (*Mtu*) RecX, are middle size proteins (171- to 188-residue long polypeptides) that share a significant degree of identity (>40%, ClustalW2 alignment) among them. RecX*_Mtu_* shares a higher degree of identity with a large (e.g., RecX, ∼20%) than with a small RecX (e.g., RecX*_Eco_*, ∼11%) protein, with RecX and RecX*_Eco_* sharing a very low, ∼15%, overall identity (Figure S1).

RecX of the Firmicutes Phylum (83 orthologues analyzed), represented by *B. subtilis* RecX (*Bsu*, a 264-residue long polypeptide), are large proteins (212- to 272-residue long polypeptides) that share a significant degree of identity (>30%) among them. Inspection of the genetic organization around the Firmicutes *recX* gene, however, discards any conservation on the genome context, even within the closely related Classes of the Phylum. Examination of the amino acids sequence of RecX342 revealed that the conserved L101 (encircled) of the predicted α-helix 3 on repeat 1 (R1 α3, [63]) was substituted by P (L101P) (Figure S1).

From these data altogether it could be assumed that the small, middle and long proteins are distantly related classes that perform a similar function, and that longer proteins might have an additional uncharacterized function at the C-terminal region. However, secondary structure prediction of Firmicutes RecX revealed that the C-terminal region, which shares no significant identity with any domain of known activity, might fold as three tandem α-helix motifs. Examination of the amino acids sequence of the 43 C-terminal residues of RecX (residues 221–264) revealed significant identity with an internal region of few RecX orthologues (e.g., *Provotella oulorum*, 38% 15/40 residues). In *P. oulorum* RecX, this region aligned with the region of RecX*_Eco_* that forms the R3α2 and R3α3 motifs (data not shown), suggesting that Firmicutes RecX, which seems to lack R1α1 and R1α2 helices when compared to RecX of different Phyla (Figure S1), might also consist of three tandems repeats of a three-helix motif.

### The absence of RecX reduces the threshold for SOS response

*In vivo* analyses of *B. subtilis* cells revealed that: i) in response to DNA damage, RecA-dependent autocleavage of LexA triggers the SOS induction reviewed in [37], ii) expression of *recX* gene is independent of MMC-induced SOS response [64], iii) *recA* promoter utilization is reduced and delayed in *recF*15, *recO*16 or *recR*13 cells upon MMC addition [40], and iv) the interstrand crosslinks produced by MMC, as most lesions, are removed by nucleotide excision repair (NER) prior to DNA replication, but unrepaired damage induces the SOS response and then repair includes translesion synthesis and HR [65]. To determine whether a *recX* mutation has an effect on the levels of SOS response, the *rec* ^+^, Δ*recX*, *recX*342 or Δ*lexA* cells were exposed to increasing MMC concentrations, the cultures were harvested 30 min later and the levels of RecA protein, expressed from its native locus and promoter, were measured. Equivalent amounts of crude extracts proteins were separated by SDS-PAGE, transferred and blotted against polyclonal antibodies raised against RecA. Serial dilutions of purified RecA were used as concentration standard.

**Figure 2:**
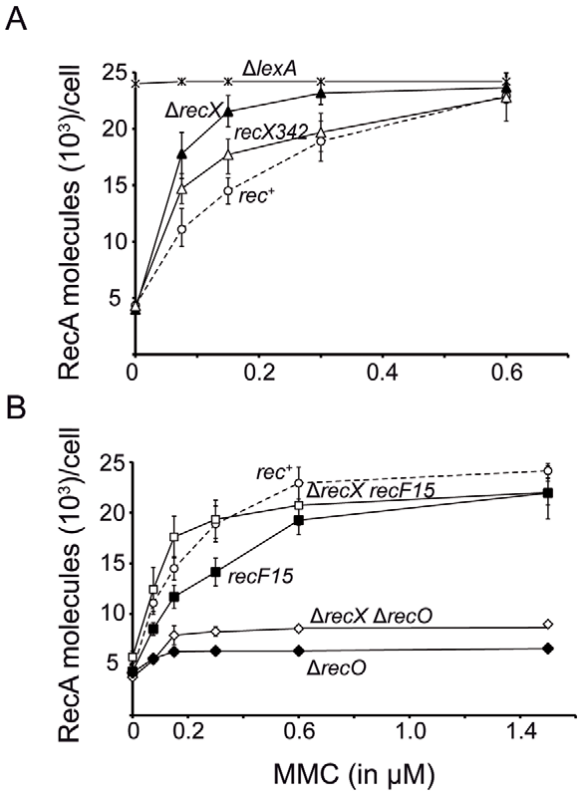
*B. subtilis* RecA protein accumulation upon SOS induction in different genetic backgrounds. Cells were grown to OD_560_ = 0.4 in LB medium and exposed to increasing concentrations of MMC for 30 min. The cells were lysed and equivalent protein amounts subjected to 10% SDS-PAGE, followed by immunoblot transfer (see Materials and Methods). (A) *rec* ^+^, Δ*recX*, Δ*lexA* and *recX*342 cells; (B) *rec* ^+^, *recF*15, Δ*recX recF*15, Δ*recO* and Δ*recX* Δ*recO* cells. The results are the average of at least four independent experiments and the standard errors are indicated.

The absence of RecX (Δ*recX*) or the presence of the RecX342 or RecF15 variants did not affect the basal level of RecA when compared with *rec* ^+^ cells (estimated to be ∼4,500 monomers per cell, or ∼6 µM assuming an average cell volume of 1.2 fL) (Figure 2A and 2B). The absence of RecO, however, slightly reduced it, and the absence of LexA rendered constitutive RecA levels (Figure 2A and 2B).

The RecA protein reached its maximal level at ∼0.6 µM MMC, and maximal induction caused 4- to 6-fold increase in net RecA in the *rec* ^+^ context [66], Figure 2A. As expected, in the absence of the LexA repressor, the level of RecA was comparable to levels detected upon full SOS induction (≥0.6 µM MMC) in the *rec* ^+^ context (Figure 2A). In the absence of RecX, a significant net RecA accumulation (∼18,000 monomers per cell) was observed upon exposure to MMC concentrations as low as 0.07 µM. Similar results were observed in the *recX*342 context (Figure 2A). This reduced threshold for SOS response upon MMC addition in *recX* could be attributed to the lack of negative regulation of RecA. This is consistent with the observation that 0.07 µM MMC concentration neither compromised cell proliferation nor cell plating efficiency in the *rec* ^+^ and Δ*recX* context (data not shown). Furthermore, this SOS induction did not significantly contribute to error-prone repair by translesion synthesis (Text S1, Annex 1) in the Δ*recX* context.

### RecF and RecX contribute to RecA filament formation

*In vitro* studies revealed that: i) RecX*_Eco_* blocks RecA*_Eco_* filament extension reviewed in [22], and ii) RecX*_Eco_* physically interacts with RecA*_Eco_* and RecF*_Eco_* [29], [67]. To test the effect of the absence of the RecX and RecF functions in SOS response, the levels of RecA were measured 30 min after MMC addition. The *recF*15 mutation reduced net RecA accumulation when compared to *rec* ^+^ cells and higher concentrations of MMC were needed to reach full induction. The absence of RecX reversed the effect of the *recF*15 mutation on the level of RecA, with RecA levels comparable to *rec* ^+^ cells (Figure 2B). It is likely that: i) the activation of RecA as a coprotease (RecA filamented onto ssDNA), to facilitate self-cleavage of LexA, is modulated by RecX and RecF in response to MMC addition; ii) in the absence of RecX and RecF there is not net change in RecA induction (RecA·ssDNA filament formation), with one counteracting the activity of the other; and iii) the RecA filaments formed in the absence of both RecX and RecF are sufficient for SOS response (Figure 2B), but are not proficient for RR (Figure 1B) and GR (Table 2), suggesting that both modulators are necessary to avoid potential hazards that could be caused by miss-regulation of HR.

**Table 2:** RecX alone, or in concert with RecO or RecF, plays an important role in chromosomal and plasmid transformation.

Strain	% Chromosomal transformation^a^	% Plasmid Transformation^b^
*rec* ^+^	100 (5.7×10^−3^)	100 (4.7×10^−5^)
*recF15* c	71.4	96.2
Δ*recR* c	69.0	94.1
Δ*recO* c	58.2	3.2
*recX*342^c^	60.9	93
*recX342* Δ*recO* c	<0.1	0.1
*recX342* Δ*recR* c	<0.1	0.9
*recX342 recF15* c	<0.1	<0.1
Δ*recA* c	<0.01	97.4
Δ*recX*	0.5	1.8
Δ*recX* Δ*recA*	<0.01	57
Δ*recX* Δ*recO*	<0.1	<0.1
Δ*recX recF15*	<0.1	<0.1

The *metB*5 locus contains a single point mutation.

a The yield of *met* ^+^ transformants (SB19 DNA, chromosomal transformation) and

b kanamycin resistant transformants (pUB110, plasmid transformation) was corrected for DNA uptake and cell viability, and the values obtained normalized relative to that of the *rec* ^+^ strain, taken as 100. Between parentheses is included the number of transformants/cell.

c The transformation frequencies of *rec* ^+^, *recF*15, Δ*recR*, Δ*recO*, *recX342* (previously termed *recH*342), *recX342* Δ*recR*, *recX342 recF*15, *recX342* Δ*recO*, *recX342* Δ*recR* and Δ*recA* cells were reported elsewhere [13], [15], [16], [52] and determined here for direct comparison. The results are the average of at least three independent experiments and are within a 10% standard error.

In the absence of RecO addition of MMC slightly increased (∼1.2-fold) the RecA levels when compared to the non-induced control (Figure 2B). The absence of RecX slightly increased the RecA levels in the Δ*recO* context (Figure 2B). It is likely that in the absence of the RecO mediator there was a marginal increase in the nucleation of RecA protein filaments, and the RecX modulator poorly contributed, if at all, to RecA nucleation in the Δ*recO* context (Figure 2B).

### Damage-induced RecX foci

**Figure 3:**
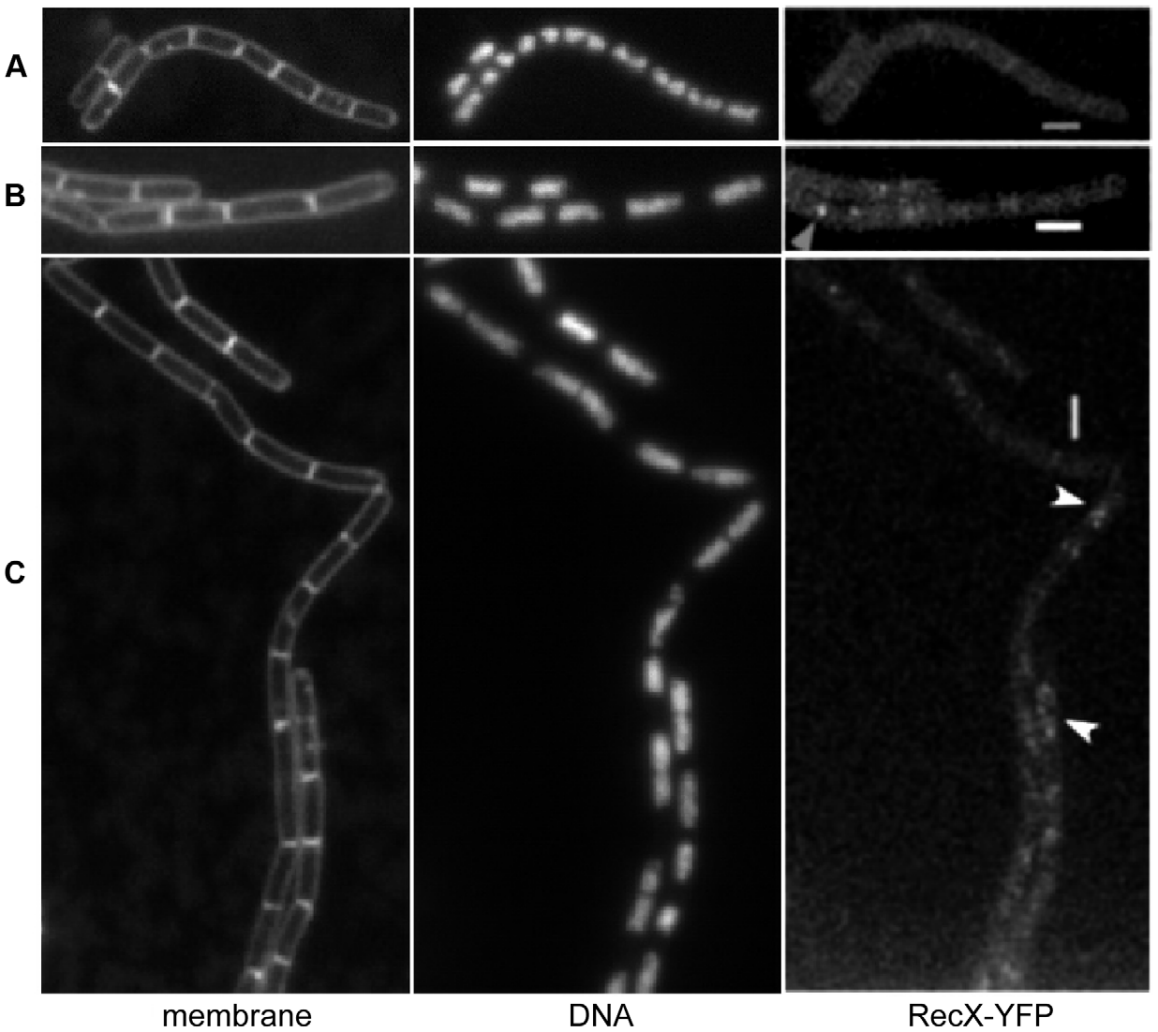
Fluorescence microscopy of growing *B. subtilis* cells expressing RecX-YFP. (A) RecX-YFP in exponentially growing cells. (B and C) Cells at 60 (B) and 180 min (C) after addition of 0.15 µM MMC. Shown are membrane stain, DNA and the corresponding RecX-YFP fluorescence. White arrowheads on the overlay denote the few RecX foci visible 180 min after MMC addition. White bars 2 µm.

In response to DNA damage RecO, RecR and RecA form foci at 30 to 45 min, followed by RecF at 60 min after damage [31], [32]. To gain insight onto the mechanism by which RecX modulates RecA functions, RecX was visualized in cells grown in minimal medium at 25°C (Figure 3). A strain bearing a C-terminal fusion of RecX to YFP (RecX-YFP) was constructed (Text S1, Table S1). The RecX-YFP fusion, integrated in its native locus was fully functional. The growth rate (data not shown) and the observed survival curve, upon acute exposure to increasing concentrations of MMC for 15 min, of *rec* ^+^ and r*ecX-yfp* cells were similar. As observed with other DNA damaging agents (Figure 1), the Δ*recX* strain was moderately sensitive to varying concentrations of MMC, and the *recF*15 Δ*recX* was extremely sensitive (Figure S3).

Microscopic observation of the strain in exponential growth revealed dispersed localization of RecX-YFP throughout the cells (Figure 3A), whereas this pattern of localization changed dramatically upon MMC (0.15 µM) addition. In ∼40% of the cells, RecX was concentrated into distinct foci on the nucleoid (mostly two per cell, but sometimes up to 5, 300 cells analyzed) 60 min after the addition of MMC (Figure 3B), and in 52% after 120 min (300 cells analyzed) (data not shown). Upon DNA damage, RecX was localized as distinct foci after RecA-induced foci formation, which suggests that RecX acts after RecO, RecR and RecA, and concomitant with RecF. The number of RecX foci decreased (Figure 3C) 180 min after addition of MMC, until foci were no longer detectable, and growth of cells slowly resumes.

### RecX co-localizes with RecA threads

**Figure 4:**
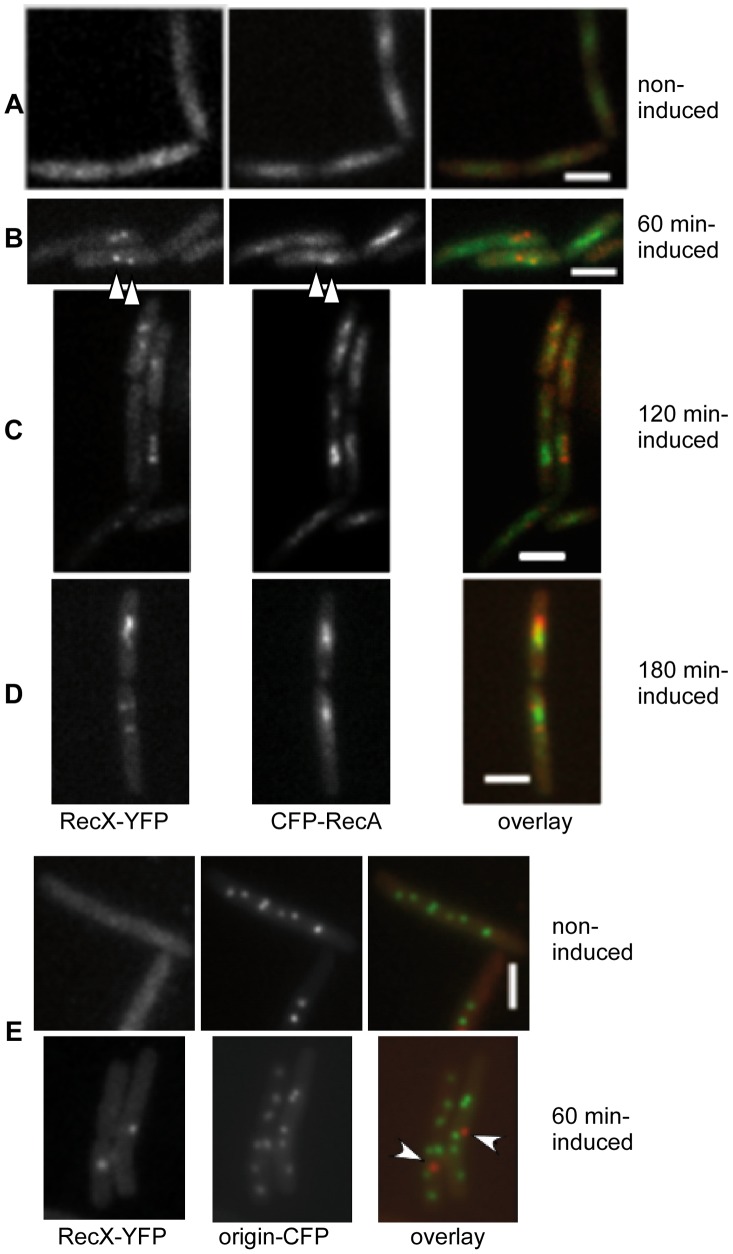
*B. subtilis* RecX-YFP has a similar subcellular position to CFP-RecA threads and does not co-localize with sites of DSBs. (A) RecX-YFP, CFP-RecA and overlay in exponentially growing cells. (B–D) Cells at 60 (B), 120 min (C) and 180 min (D) after addition of 0.15 µM MMC. Shown is the corresponding RecX-YFP or CFP-RecA fluorescence, and an overlay of both signals (RecX in red, RecA in green). White triangles indicate examples of colocalization at 60 min. (E) Fluorescent microscopy of cells during mid-exponential growth (upper panels) or after a defined break (lower panels). After induction of HO endonuclease cutting close to origin regions decorated with LacI-CFP (60 min induced), RecX foci (white arrows on the overlay) generally do not coincide with the cut sites. White bars 2 µm.

Since biochemical [29], [38], [39] and structural analysis [63], [67] have shown that RecX*_Eco_* interacts with the RecA*_Eco_*·ssDNA filament, we set out experiments to visualize both proteins in living cells (Figure 4). Cells bearing a N-terminal fusion of RecA to CFP (CFP-RecA) were previously described (Text S1, Table S1) [32]. Microscopic observation of the strain in exponential growth revealed dispersed localization of RecX-YFP throughout the cells (Figure 3A and Figure 4A), and CFP-RecA throughout the nucleoid [32], Figure 4A. In response to DNA damage CFP-RecA formed foci at 30 to 45 min, followed by RecX at 60 min. Sixty min after MMC addition the RecA foci started to be more and more condensed, and then formed highly dynamic filamentous structures (Figure 4B). The formation of dynamic thread-like structures of CFP-RecA was maximal at 120 min after addition of MMC, as well as the number of cells containing RecX-YFP foci, which generally co-localized with RecA threads (Figure 4C). From 350 analyzed cells, RecX-YFP foci localized at or near the RecA signals in 41% of the cells (that is in 91% of all cells showing RecX-CFP foci), adjacent to RecA signals in 3% of the cells, or clearly did not co-localize in 1% of the cells; 55% of the cells showed CFP-RecA fluorescence, but no detectable RecX-YFP foci. Between 120 and 180 min, CFP-RecA threads became fewer in number and thus apparently disassembled, until about 180 min, repair was terminated and RecA threads were no longer visible (Figure 4D, central panels). The number of cells containing clear RecX-YFP foci also decreased in a time dependent manner up to 180 min after induction (still co-localizing with subcellular locations of high CFP-RecA signals) (Figure 4D), after which foci declined to negligible levels (0.7% of the cells showed foci, 280 analyzed cells). Growth resumed ∼180 min after the initial DNA damage in *rec* ^+^ cells.

### RecX is not recruited to a defined DNA DSB

*In vivo* analyses revealed that: i) RecN is recruited to a defined DSB [32], [68], and ii) RecO, RecR, RecA and RecF co-localize with DNA damage-induced RecN focus [31], [32]. To test whether RecX was also recruited to a defined DSB, a strain bearing a xylose inducible promoter transcribing the HO endonuclease, an HO cleavage site and a *lacO* site, both integrated close to the *ori*C region, and the *lacI*-*cfp* cassette ectopically integrated at the threonine locus, was constructed as previously described (Text S1, Table S1) [32].

After induction of the HO endonuclease a single two-ended DNA break was induced and RecX-YFP foci were observed in about 10% of the cells (350 analyzed cells). The observed RecX foci were generally not coincident with the *oriC* (LacI-CFP) signal, only 1 out of 34 foci was coincident with an *oriC* signal (Figure 4E). These experiments show that RecX is not directly recruited to sites of DSBs.

### RecX promotes the disassembly of RecA threads

**Figure 5:**
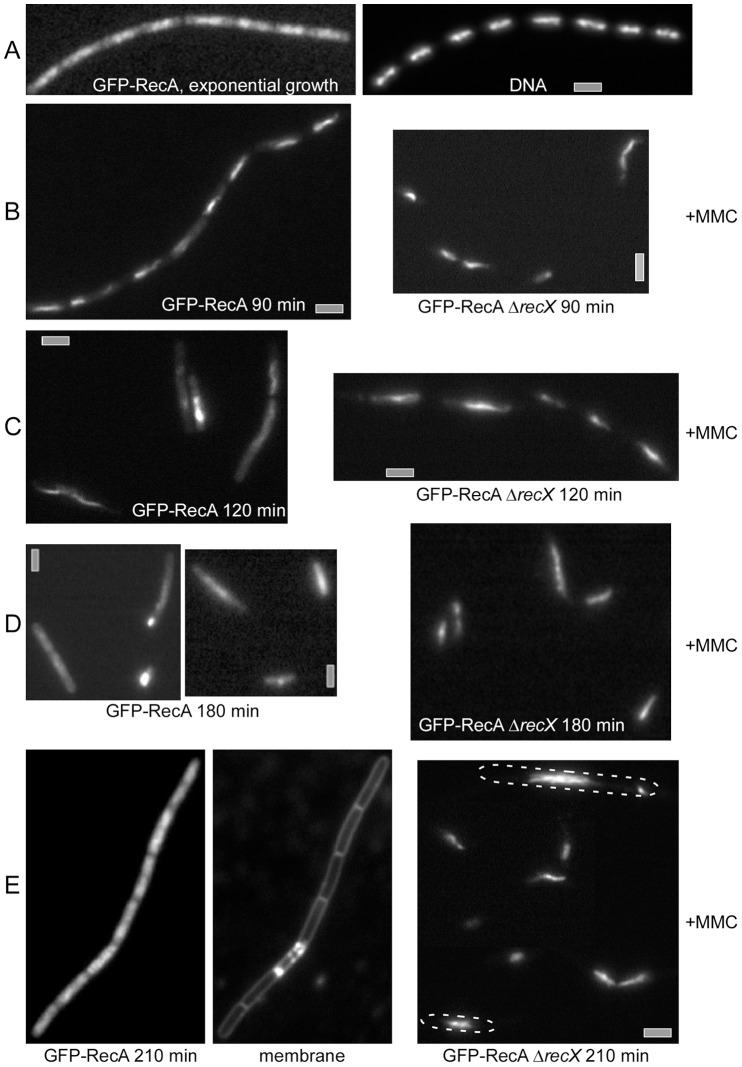
Fluorescence microscopy of *B. subtilis rec^+^* and Δ*recX* cells expressing CFP-RecA. (A) CFP-RecA in exponentially growing cells. (B–E) Cells at 90 (B), 120 (C), 180 (D) and 210 min (E) after induction of DSBs by 0.15 µM MMC. CFP-RecA threads are visible on the nucleoid (some cells whose outline is unclear are denoted by a dotted line) in Δ*recX* cells 210 min after MMC addition. Note that this panel is a composite image. White bars 2 µm.

Biochemical studies have shown that RecX*_Eco_* (RecX*_Ngo_*) blocks RecA*_Eco_* assembly onto ssDNA tracts [29], [38], [39] or facilitates a more rapid RecA*_Ngo_* filament disassembly [49]. To test whether RecX affects RecA foci formation (“nucleation”) and/or thread assembly or disassembly (“filament formation”), the localization of a functional CFP-RecA fusion in *rec* ^+^ and in the Δ*recX* strain was monitored. For all times points, 350–400 cells were analyzed. During exponential growth, RecA localized throughout the nucleoids in both, *rec* ^+^ (Figure 5A) and Δ*recX* cells (data not shown). The absence of RecX neither affected the formation of RecA foci nor the assembly of RecA threads between 60 and 90 min after induction of DNA damage (data not shown), and for the first 120 min following the addition of MMC, no obvious difference in the formation of CFP-RecA threads was detectable between *rec* ^+^ and Δ*recX* cells, 75 to 85% of the cells contained CFP-RecA threads (Figure 5B and 5C).

The number of cells showing threads decreased in *rec* ^+^ cells to less than 50% after 120 min, while 80% of all Δ*recX* cells continued to contain RecA threads (Figure 5C). After 180 min only ∼4% of *rec* ^+^ cells contained visible CFP-RecA threads (many contained CFP-RecA accumulations at a single cell pole), while these structures persisted in 95% of the *recX* mutant cells (Figure 5D). Even after 210 min, CFP-RecA threads were visible in >50% of mutant cells, while in *rec* ^+^ cells, RecA was again spread uniformly on the nucleoids, and thread structures were only observed in 1.3% of the cells (Figure 5E). These data reveal that RecX is necessary for the down-regulation of RecA threads, which most likely consist of RecA·ssDNA filaments. It is likely that the balance between RecX and RecF governs the dynamics of RecA threads, but at late times, when the DNA damage signal is removed, the thread-stabilizing activity of RecF might be negatively modulated by an uncharacterized function(s), leading to net thread-destabilizing activity of RecX.

### RecX localizes at a pole and on the nucleoid in cells grown to competence

To gain insight into the chromosomal gene transfer barriers (see Introduction), the fate of RecX during GR was analyzed. At the onset of stationary phase, only 10%–20% of cells stochastically develop time-limited competence in response to specific environmental conditions. Natural competence is a genetically programmed process with a specialized membrane-associated machinery for uptake of exogenous dsDNA that subsequently processes and internalizes ssDNA into the cytosol (DNA uptake machinery) [69]. Previously it has been shown that some soluble proteins of the recombination apparatus (namely SsbB, DprA, RecA, RecU and CoiA) are located at the cell poles, where they co-localize with the DNA uptake machinery [7]–[11]. To understand the role of RecX on GR its localization was analyzed upon competence induction. Microscopic observation of RecX-YFP in cells grown to competence revealed fluorescent foci in 8 to 10% of cells (1260 cells analyzed) (Figure S4), suggesting that this is the proportion of competent cells. In the 8–10% of the cells RecX-YFP existed mainly as one focus per cell (73% of the cases), sometimes localizing to a single cell pole (∼27%), but mostly at midcell in the nucleoid (∼46%) (Figure S4). Less often, two (∼17%) foci (mostly one at the pole and one at the nucleoid), three foci (∼5%), and patched structures (∼5% of the cases) were observed.

To investigate the nature of RecX-YFP foci, we performed time-lapse microscopy, capturing images of cells grown to competence without DNA, or 30 min after addition of DNA, with 2 s time intervals (Figure S5). Irrespective of the presence or absence of DNA, RecX-YFP foci at midcell were dynamic and moved between acquisitions (note that the signal was weak, so only few frames could be captured), while foci at or near the cell pole did not move away from their position. The total number of fluorescent cells (7% of total cells in the absence of DNA) did not change markedly after addition of DNA (to 10%), but the number of cells having one discrete focus increased from 73% to 82% of the cells containing a signal 30 min after DNA addition. With increasing time upon DNA addition (0 to 30 min) the number of RecX-YFP cells with one focus at the pole decreased (from 27% to 13%) and the number of cells with one focus on the nucleoid augmented from 46 to 72% (at least 1000 cells were analyzed for each time point) (Figure S5). Upon addition of DNA, the number of cells with more than one RecX-YFP focus and with patched structures became lower to less than 1.5% of the competent cells. Figure S5, movie A, shows a polar focus that moved around the cell pole, but remained there, movie B shows a cell with many foci that moved, and movie C shows a central focus that moved. Thus, polar foci are usually static, possibly representing RecX that is associated with the DNA uptake machinery or any associated recombination protein, and non-polar foci are very dynamic.

### RecX is important for chromosomal transformation

*B. subtilis* competent cells can take up DNA of any source and the transfer of chromosomal genes requires HR. The frequency of appearance of *met* ^+^ chromosomal transformants in single *rec*-deficient strains, classified within the α (*recF*15 or Δ*recO*), β, γ (*recX*342), δ, ε, ζ or η epistatic groups (Figure S2), does not vary more than 3-fold relative to the *rec* ^+^ value [12], [13], [15], but is blocked in the Δ*recA* context (Table 2). It is likely that a certain redundancy exists and/or that the critical functions were not studied yet. The absence of RecX severely impaired chromosomal transformation (∼200-fold) with respect to that of the *rec* ^+^ strain (Table 2).

To establish the potential contribution of RecO and/or RecF in the *recX* context, the capacity of these cells to be transformed with chromosomal DNA was measured. Chromosomal transformation was inhibited >1,000-fold in the *recF15* Δ*recX* or Δ*recO* Δ*recX* context, when compared to the absence of RecA that blocked it (>10,000-fold) (Table 2). It is likely that distinct effectual length or packing density of the RecA·ssDNA filament is necessary for chromosomal transformation (Table 2).

### The absence of RecA suppresses the *recX* impairment on plasmid transformation

In the absence of homology with the recipient DNA, a linear ssDNA oligomeric plasmid molecule requires RecO, DprA and RecU for establishment [9], [10], [15], [70], [71]. Plasmid transformation, however, was only marginally affected relative to the *rec* ^+^ strain in the Δ*recA* context or in single *rec*-deficient strains classified within the α (*recF15*, Δ*recR*), β, γ (*recX*432), δ, ε (*ruvA2*, Δ*ruvB*), ζ or η epistatic groups (Figure S2) [9], [13], [15], [72]. Plasmid transformation was markedly reduced in the absence of RecX (Table 2). Based on results described above and the observation that the RecA·ssDNA filaments might open an unproductive avenue that is deleterious for plasmid transformation [9], [71], it is hypothesized that RecX might be required to dislodge RecA from the internalized ssDNA. If the hypothesis is correct the absence of RecA should suppress the need for RecX. To test this hypothesis the Δ*recX* Δ*recA* strain was constructed. The absence of RecA partially suppressed the RecX requirement for plasmid transformation, but as expected it remained blocked in chromosomal transformation (Table 2). It is likely that the RecA·ssDNA filaments might be deleterious for plasmid transformation and RecA modulators, namely RecX (Table 2) and RecU [9], [71], are required to catalyze RecA·ssDNA filament disassembly or to block filament assembly. Alternatively, in the absence of both RecA and RecX proteins, an uncharacterized recombination pathway (specific for plasmid transformation) becomes operative. To test this hypothesis, the effect of the absence of RecX and RecO, or RecX and RecF in plasmid transformation frequencies was measured after construction of the respective strains. Both chromosomal and plasmid transformation were blocked in the Δ*recX recF* or Δ*recX* Δ*recO* context (Table 2), it was therefore considered unlikely that an uncharacterized recombination pathway exists. A similar inhibition of GR was observed in the *recF*15 *recH*342 (*recX*342) or Δ*recO recH*342 (*recX*342) strain (Table 2) [15], [52].

## Discussion

The *recH*342 mutation (γ epistatic group), which leads to decreased interspecies gene transfer, maps in the putative *recX* gene (Table 1). The absence of RecX renders cells deficient in RR, hence *recX* was considered a genuine recombination gene, and the *recH*342 mutation was renamed as *recX*342. The classification of Δ*recX* mutation within the epistatic group γ was confirmed by combination of the Δ*recX* mutation with representative members of different epistatic groups (Figure S2, Text S1, Annex 2). As expected from data derived with *recX*342 [52], *recX* was neither epistatic with *recF*, *recO* (Figure 1) and *recR* (α), *addA*, *addB* (β), *recN* (δ), *recU*, *ruvAB* (ε), *recJ*, *recS* and *recQ* (ζ), *recG* (η epistatic group) nor with *ku* (a function implicated in non-homologous end-joining) (C.E.C., G. Garaulet, C. Marchisone and J.C.A., unpublished results).

### RecX antagonizes RecF during the SOS response

In all systems with a genuine SOS response system, RecA needs to be recruited onto SSB-coated ssDNA tracts at a blocked replication fork reviewed in [19], [22]. We show that in the absence of RecX (presence of RecO and RecF), low doses of MMC, which did not significantly affect the cell doubling time, are sufficient to induce the SOS response (Figure 2A). In contrast, in the absence of RecO, high lethal concentrations of MMC were needed to marginally induce RecA synthesis when compared to *rec* ^+^ cells, and in the absence of both, RecO and RecX, the synthesis of RecA was marginally facilitated when compared to Δ*recO* cells (Figure 2B). It is likely that: i) RecO is essential for RecA nucleation and SOS induction, and the absence of RecX cannot override such defect; ii) RecF [31] and RecX (Figure 3 and 4) act after RecO as deduced from the cytological studies; and iii) the RR and GR deficiency in the Δ*recO* Δ*recX* context could be attributed to decreased RecA·ssDNA nucleation and filament dynamics. This is consistent with the *in vitro* observation that RecO (RecOR*_Eco_*) protein(s) contribute to RecA (RecA*_Eco_*) filament nucleation onto SsbA- (SSB*_Eco_*)-coated ssDNA and that RecOR*_Eco_* is unable to counteract the inhibitory effects of RecX*_Eco_* on RecA*_Eco_* filaments [22], [29].

In the absence of RecF (presence of RecX and RecO) high lethal concentrations of MMC were needed to marginally induce RecA synthesis when compared to *rec* ^+^ cells. In the absence of both RecA modulators (RecX and RecF), however, the levels of RecA induction showed a profile similar to *rec* ^+^ cells (Figure 2B). It is likely that there is a cross talk among RecX and RecF and one might antagonize the effect of the other, and that RecO and RecF contribute to RecA filament formation at different stages (Figure 2B). Conversely, in *E. coli* the formation of RecA filaments decreased in the Δ*recX* strain, and this RecX-mediated destabilization of the RecA·ssDNA filaments was independent of RecFOR [30], [38], [73], and overexpression of RecX [38] or RecF [74] inhibited induction of the SOS response after UV irradiation.

The RecA·ssDNA filaments formed in the *recF*15 Δ*recX* context are sufficient for SOS induction, but are not proficient for HR (Figure 1 and Table 2). It is likely, therefore, that there are different layers of regulation of RecA·ssDNA filament extension with RecF and RecX acting at different levels, and as biological antagonists. How can we rationalize this observation? It is likely that there are different types of RecA·ssDNA filaments. In the Δ*recX* context, a high local RecA concentration may generate a saturated RecA·ssDNA filament, resulting in a higher affinity for LexA. In the *rec* ^+^ or Δ*recX recF*15 cells, sub-saturated RecA·ssDNA filaments may be formed at low MMC dose, and in these conditions RecA equilibrates among the existing DNA lattices lowering its affinity for LexA. Here, a higher MMC dose was required to induce the SOS response when compared to the Δ*recX* context. Alternatively, the length of the RecA·ssDNA filament, rather than the packing, is a crucial factor in the rate-limiting step of homologous pairing [34], [49].

### RecX affects the formation of RecA threads during recombinational DNA repair

DNA damage-induced RecA formed discrete foci on the nucleoid ∼30 min upon MMC addition [32]. RecX formed discrete foci on the nucleoid and a RecA focus started to be converted into irregular RecA threads after 60 min of MMC addition (Figure 4). RecA thread formation was demonstrated to be independent of RecF [31], [32] and RecX (Figure 4 and Figure 5). Within 60–120 min upon DSB induction the RecX-YFP foci increased in number, and the RecA threads were dynamic filamentous extensions, to dissociate from the DNA and become undetectable at about 180 min in *rec* ^+^ cells grown in minimal medium at 25°C (Figure 5). In the absence of RecX, RecA threads persisted for an extended period of time (Figure 5), revealing that RecX affected the dynamics of RecA threads (RecA·ssDNA filament disassembly) rather than RecA foci (RecA nucleation) and thread formation (RecA·ssDNA filament extension). Conversely, in response to UV irradiation the formation of *E. coli* RecA foci decreased in the Δ*recX* or *recF*4115 context [30]. Unlike the RecO, RecA and RecF foci, which co-localize with RecN and with HO endonuclease-generated DSBs, the RecX foci did not co-localize with DNA DSBs (Figure 4). Consistent with its activity on RecA threads, RecX formed foci after RecA nucleation and co-localized at or near RecA threads (Figure 4). We propose that RecX has two activities: to counteract the activity of RecF, and to mediate RecA·ssDNA filament dislodging. These activities are essential for rendering a dynamic balance between RecA assembly and disassembly from ssDNA, in order to form an “active RecA·ssDNA filament” and to facilitate the inherent DNA pairing activity of the formed filaments during the search for homology.

### RecX plays a RecA-dependent function in genetic recombination

Plasmid transformation is a RecA-independent process [72]. It was previously postulated that RecA-bound to the incoming oligomeric linear plasmid ssDNA was deleterious [71]. If the working hypothesis is correct, the RecA·ssDNA filaments should be disassembled, either by RecX (Table 2) or by RecU [71], for plasmid transformation. We have shown that RecX is necessary for plasmid transformation, and such requirement is overcome by the deletion of *recA* (Table 2), rather than by the opening of a new and uncharacterized recombination avenue.

During natural transformation donor DNA enters the cell as ssDNA molecules, hence end processing is not necessary. Chromosomal transformation is strictly dependent on the presence of a fully functional RecA·ssDNA filament to catalyze intermolecular recombination between the incoming ssDNA and the homologous duplex recipient DNA without significant DNA synthesis [12], [75]. Competence-induced RecX formed discrete foci at the entry pole and on the nucleoid in the absence of DNA. Upon addition of dsDNA, SsbB (in concert with SsbA) at the entry pole protects the incoming ssDNA and limits RecA loading onto SsbA- and/or SsbB-coated ssDNA [18]. RecO (or DprA) displaces SsbA and/or SsbB and recruits RecA onto SsbA- and/or SsbB-coated ssDNA leading to RecA foci formation at the entry pole [8]–[10], [18]. RecX at the entry pole and in the nucleoid should modulate RecA thread formation (RecA·ssDNA filament extension). RecA forms a filament (thread) extending from the pole to the centrally located nuclear body [8]. A RecA thread might facilitate the search for a homologous segment and mediate joint molecule formation (heteroduplex) with the resident chromosome [9]. The frequency of chromosomal DNA transformation marginally decreased in the *recX*342 background, but dropped to low levels (∼200-fold) in the Δ*recX* context (Table 2). Studies in other bacteria are less clear, because the absence of RecX*_Dra_*, results in elevated chromosomal transformation frequencies (∼2.5-fold increase) [76], whereas the absence of RecX*_Ngo_* also decreases recombination frequencies, although the effect was modest (∼5-fold reduction) [60]. *In vitro* RecX*_Ngo_* destabilizes RecA*_Ngo_*·ssDNA filaments by causing its local instability [49]. Although *B. subtilis* and *N. gonorrhoeae* RecX proteins might have similar functions, there are also important differences. For example, in the latter species the presence of RecF is not obvious [53], suggesting that dynamic RecA·ssDNA filament formation is modulated in this bacterium by different partners than those found in *B. subtilis* cells.

Cohan and coworkers [6], [77] reported that the frequency of unidirectional transfer of DNA between donor and recipient (chromosomal transformation) in *B. subtilis rec* ^+^ cells decreases with increased sequence divergence. Here, each 5% increment in sequence divergence yields ∼ a 10-fold decrease in chromosomal transformation [6], [77]. The *recX*342 (BG119) competent cells showed a >20-fold increased sensitive to sequence divergence than when transformed with homogamic DNA [6]. However, there is no measurable effect in preventing inter-species transformation in the *recF*15 (BG129) or *recO*16 (BG107) context. Furthermore, the frequency of intra- or inter-species transformation dropped to undetectable levels in the Δ*recX recF*15 or *recX*342 *recF*15 context (Table 2), leading to populations with a clonal structural potential. We propose that the RecA·ssDNA filament forms a metastable reversible intermediate, whose dynamic modulation is governed by RecF and RecX and predict that the RecX342 variant makes recombination initiation (and also termination) very sensitive to sequence divergence. A RecA·ssDNA filament to initiate recombination between donor and recipient DNA requires a MEPS of 25- to 35-base pairs to initiate recombination between donor and recipient DNA [78]. A similar extent of sequence identity was reported for viral-mediated plasmid transduction [79], [80]. It is likely that the effectual “length/packing” of the RecA·ssDNA filament and the MEPS needed for proficient inter-specific recombination are more stringent than for intra-specific recombination, and that RecX and RecF prevent the dissociation of potentially unstable heteroduplex intermediates that are essential for HR (Figure 1 and Table 2).

### Model for the formation of an effectual RecA·ssDNA filament

The RecX*_Eco_* protein contributes modestly to recombinational potential reviewed in [22]. *In vitro*, the ability of small RecX (<180 residue long, *e.g.*, RecX*_Eco_*) to inhibit RecA*_Eco_*-associated activities, *i.e.* ATPase, strand-exchange, and LexA*_Eco_* cleavage activities [38], [63], [67], have prompted different groups to categorize RecX*_Eco_* as a major negative regulator of RecA*_Eco_* activities. The current model postulates that RecX*_Eco_* bound to the deep helical groove of the RecA*_Eco_* nucleofilament blocks RecA*_Eco_* assembly onto ssDNA, leading to filament destabilization and inhibition of HR. Based on previous data reviewed in [19] and the ones presented here, we propose that RecX is necessary to avoid potential hazards that could be caused by miss-regulation of HR.

**Figure 6:**
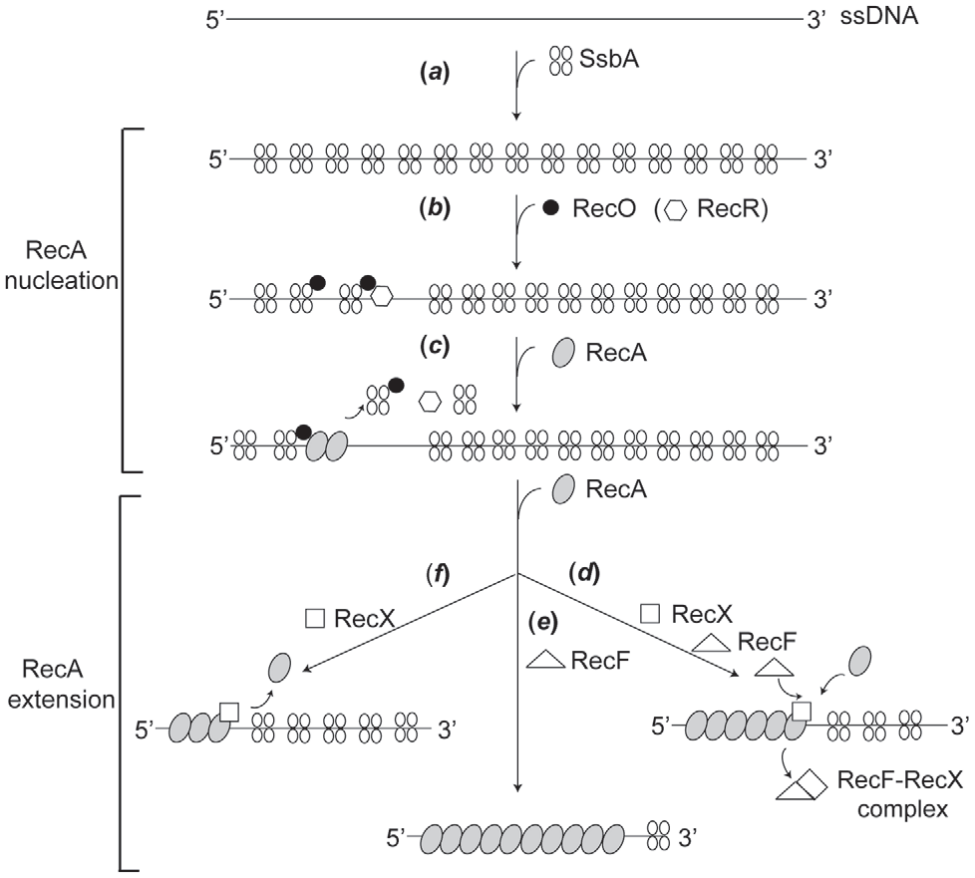
Dynamic *B. subtilis* RecA assembly into SsbA-coated ssDNA. It is proposed that RecO (perhaps in concert with RecR) mediates RecA nucleation, and RecF and RecX modulate RecA filament extension. SsbA binds to ssDNA and limits RecA nucleation (step *a*). SsbA recruits RecO (RecOR*_Eco_*) onto SsbA-coated ssDNA. RecO might recruit RecR. RecO interacting with ssDNA promotes limited dislodging of SsbA, and facilitates RecA nucleation (step *c*). RecF and RecX lead to an effectual RecA filament (step *d*). In the absence of RecX, the contribution of RecF on RecA nucleation and/or filament extension is poorly understood (step *e*). In the absence of RecF, RecX facilitates the dynamic disassembly of the RecA filaments (step *f*).

We propose that SsbA, at the ssDNA in a stalled replication fork, interacts with RecO and loads it (or RecOR) onto SsbA-coated ssDNA (Figure 6, step b) [70]. Then, RecO loads few RecA*_Eco_* monomers onto ssDNA with a limited displacement of SsbA and/or SsbA·RecO (RecR) complexes (Figure 6, step c) [47]. The role of RecF in RecA nucleation is unclear, because RecF focus formation is impaired in the absence of RecO [31], and DNA damage-induced RecF foci, which co-localize with a RecA focus, are clearly detected after RecA focus formation, and concomitantly with RecA thread formation and RecX foci formation (Figure 5) [31], [32]. In *E. coli* cells, RecOR or RecFOR, upon interacting with SSB, loads few RecA monomers onto ssDNA with a limited displacement of SSB [46], [81], [82], [83].

Regulation of the cycle of the assembly and disassembly of RecA is achieved through the hydrolysis of (d)ATP, whereas the extension (formation of RecA threads) is controlled by RecA modulators (*e.g*., RecX, RecF, RecU). Based on the data presented here and the *in vitro* data of Lusetti and co-workers [29] we propose that there might be a crosstalk between RecF and RecX in the modulation of the RecA·ssDNA filament extension (Figure 6, step *d*). In the absence of RecX, RecF directly or indirectly could either facilitate RecA·ssDNA filament assembly or slow down disassembly, facilitating RecA·ssDNA filament formation even in the presence of a low DNA damage signal (Figure 6, step *e*). These “long” and/or saturated RecA·ssDNA filaments lead to premature induction of the SOS response at low-dose exposure of MMC (Figure 2), but these RecA·ssDNA filaments are neither proficient for RR nor for GR (Figure 1, Table 2). Conversely, in the absence of RecF, RecX directly or indirectly promotes RecA·ssDNA filament disassembly or delays filament assembly, leading to “short” and/or sub-saturated RecA·ssDNA filaments (Figure 6, step *f*). In the absence of both RecX and RecF, however, there is no apparent net change in the “activation” of the RecA·ssDNA filament for SOS induction, but RecA-mediated DNA strand exchange (RR and GR) is markedly impaired. We favor the view that RecX and RecF, by fine-tuning of the dynamic assembly/disassembly of RecA, facilitate the accumulation of a RecA·ssDNA filament with an effectual length or packing, as long as the DNA damage signal is on. *In vitro*, RecX*_Eco_* destabilizes RecA*_Eco_*·ssDNA filaments by either preventing growth of the filament [39] or by causing its local instability [63], and RecF*_Eco_* protects RecA*_Eco_* assembly by antagonizing the negative modulator RecX*_Eco_*, specifically during the extension phase [29]. However, *in vivo* the number of RecA*_Eco_* foci decreased in Δ*recX_Eco_*, but increased in the *recF_Eco_* context [30].

## Materials and Methods

### Bacterial strains and growth conditions

*B. subtilis* strains and plasmids used in this work are listed in Table S1. All strains were isogenic to BG214 or PY79 and were grown at 37°C on LB rich medium, unless otherwise indicated. *E. coli* XL1-Blue cells (Stratagene) were used for routine cloning. Cells were grown in Luria Bertani (LB) medium supplemented with ampicillin (100 µg ml^−1^ Amp) or chloramphenicol (30 µg ml^−1^ Cm) as required.

All enzymes were purchased from New England Biolabs. Oligonucleotides were ordered from Sigma-Aldrich. To construct the RecX-YFP fusion, the C-terminal region of the *recX* (also termed *yfhG*) gene was amplified by PCR and cloned into a plasmid carrying a downstream *yfp* gene. The resulting plasmid was then transformed into a PY79 strain, where it integrated at the original gene locus by single-crossover integration. The Δr*ecX* strain was constructed by inserting the *six-cat-six* cassette at the 5′-end of the *recX* gene as described [59]. In short, oligonucleotides pairs CGGATATCGGATCATCTGG and GTAATCGTTAAGCCTATGGATG and CGACAGCCATTGGACATATGTC and GATAGATATCGCCATCAGCCCAAG were used to PCR amplify with Vent polymerase (New England Biolabs) fragments spanning a region 721-bp and 719-bp upstream and downstream from *recX*, respectively, and overlapping at the start of the *recX* sequence. *Stu*I digestion of theses fragments, followed by ligation resulted in an *Eco*RV fragment which contains a deletion involving the 5′ end of *recX*, and that can be cloned into the same site of a pGEM-T vector (Promega), giving rise to pCB788 plasmid. *StuI*-cleaved pCB788 was joined to the *six:cat:six* cassette from vector pCB266 to give rise to plasmid pCB789. For the construction of strain BG1029, pCB789 was linearised with *Not*I and transformed into competent *B. subtilis* cells. Double mutants were constructed by transformation of the isogenic *rec*-deficient *B. subtilis* cells (*recF*15, Δ*recO*) with linear pCB789 DNA with selection for Cm^R^ (Text S1, Table S1). The *cat* gene was deleted by β-mediated site-specific recombination to render the BG1065 strain (Text S1, Table S1). The Δ*recA* mutation was introduced into BG1065 cells (Δ*recX*) by SPP1 transduction [79]. *B. subtilis* competent cultures were obtained as described previously [12].

### Sequencing of the *recH*342 strain

Genomic DNA from the Reference *B. subtilis rec* ^+^ (BG214) strain and the Test *recH*342 (BG119) strain were sequenced by high-throughput sequence analyzer (Illumina) technology using standard sequencing libraries and filtered sequence data (BGI), of ∼1 gigabases per sample, were used to conduct paired-end nucleotide sequencing with the *rec* ^+^ BG214 and the BG119 sample as described [84].

### Survival studies and DNA transformation assays

Acute survival assays were performed as previously described [66]. Briefly, *B. subtilis* cells were grown to an OD_560_ = 0.4 at 37°C in LB broth, and exposed to different concentrations of MMS, H_2_O_2_ or MMC. After 15 min (with MMS or H_2_O_2_) or 30 min (with MMC) exposition, cells were diluted and plated on LB agar plates. Colony forming units (cfu) were counted and plotted against the concentration of damaging agents, in order to obtain survival curves.

For DNA transformation experiments, *B. subtilis* competent cells were transformed with 100 ng of either SB19 chromosomal DNA to *met^+^* or pUB110 plasmid DNA to kanamycin resistance (Km^R^). Transformants were plated on minimal medium agar plates containing tryptophan but lacking methionine or on LB agar plates containing Km (5 µg ml^−1^). Transformation efficiencies were normalized to the number of viable cells plated on rich medium without selection, and the values obtained were normalized against those obtained for *rec^+^* cells.

### RecA protein expression

*B. subtilis* strains were grown in LB to an OD_560_ = 0.4 at 37°C with agitation. Then, cells were treated with increasing concentrations of MMC for 30 min. The cells were centrifuged, resuspended in buffer A (50 mM Tris HCl [pH 7.5], 300 mM NaCl, 5% glycerol) and lysed by sonication. Extracts containing equal concentrations of protein from each induction experiment alongside purified RecA protein standard (10 to 500 ng) were separated on 10% sodium dodecyl sulfate-polyacrylamide gel electrophoresis.

Polyclonal rabbit antiserum was raised against purified RecA protein according to standard protocols. Immunoblots were transferred and probed with anti-RecA antibodies as described previously [66]. RecA protein bands on developed immunoblots were quantified with a scanning densitometer (Quantity One software). Purified RecA protein standards yielded a linear relationship between antibody signal and the RecA protein concentration. The amount of RecA protein in each induced sample was interpolated from the purified RecA protein standard curve.

### Fluorescence microscopy of *B. subtilis* cells

Exponentially growing cells were obtained by inoculating overnight cultures in fresh S7 minimal media and grown to an OD_560_ = 0.4 at 37°C. Cells were then fixed with 2% formaldehyde, 4′,6′-diamino-2-phenylindole (DAPI) (1 µg/ml) was added for nucleoid visualization, and the cells were analyzed by fluorescence microscopy as previously described [31]. Mid-log phase cells were either untreated or exposed to 0.15 µM MMC for variable time and then fixed as described above. To further investigate the *in vivo* function of RecX in HR, we visualized RecX-YFP or CFP-RecA in living cells as previously described [31], [32].

## Supporting Information

Figure S1 Multiple sequence alignment of representative RecX homologues. Dashes represent gaps introduced to optimize sequence alignment. Abbreviations, accession numbers and Phylum are indicated: *Escherichia coli* (*Eco*, A7ZQC76, γ-Proteobacteria), *Pseudomonas aeruginosa* (*Pae*, P37860, γ-Proteobacteria), *Neisseria gonorrhoeae* (*Ngo*, Q5F7W3, β-Proteobacteria), *Mycobacterium tuberculosis* (*Mtu*, P0A5U8, Actinobacteria), and *Bacillus subtilis* (*Bsu*, 031575, Firmicutes). RecX sequences were aligned using ClustalW2. The position of the three α-helices for each of the three repeats (R1 to R3), derived from the *E. coli* RecX X-ray structure, are marked as rectangles above the amino acid sequence. An α-helix is split when there is an insertion that carries potential α-helix breakers. The wild type residue, RecXL101, is encircled, and the residue presented in the RecX342 strain indicated. The color code for the amino acids is the one defined by the ClustalW2 program. The fully conserved residues are framed. Colons and periods, respectively, indicate the strong and weak similar residues. The polypeptide length is indicated, and between parenthesis the full-length.
(TIF)

Figure S2 Schematic grouping of *B. subtilis* RecA-dependent DNA recombinational repair genes into different epistatic groups (α to η). Since a *recA* mutation is epistatic with any representative mutation of the different epistatic groups, it was placed in the center. The *recX* (*recX*342) mutation was placed within the γ epistatic group.
(TIF)

Figure S3 The *B. subtilis recX-yfp* fusion is fully functional. Cells were grown to OD_560_ = 0.4 in LB medium and exposed to increasing concentrations of MMC for 30 min. The strains used are indicated by the relevant mutant phenotype. The *recX-yfp* strain bears a *recX-yfp* fusion gene. The results are the average of at least four independent experiments and the standard error is indicated.
(TIF)

Figure S4 Fluorescence microscopy of *B. subtilis* cells grown to competence expressing RecX-YFP. (A) Cells expressing RecX-YFP in the absence of DNA with a focus in the nucleoid. (B) Cells expressing RecX-YFP in the absence of DNA with two foci. White bars 2 µm.
(EPS)

Figure S5 Timelapse Microscopy of *B. subtilis* cells grown to competence expressing RecX-YFP. (A) Cells with a single focus after addition of DNA. (B) cells with two foci. (C) cells with more than two foci.
(TIF)

Table S1 Strains used in this study.
(DOCX)

Text S1 Annex 1. The absence of RecX does not increase the spontaneous mutation rate. Annex 2. *recP*149 mutation maps is the *recA* gene.
(DOCX)
