# Supplementary figures and images for: RecX Facilitates Homologous Recombination by Modulating RecA Activities
Source: PLoS Genet. 2012 Dec 20;8(12):e1003126. doi: 10.1371/journal.pgen.1003126 (PMC3527212; doi:10.1371/journal.pgen.1003126)

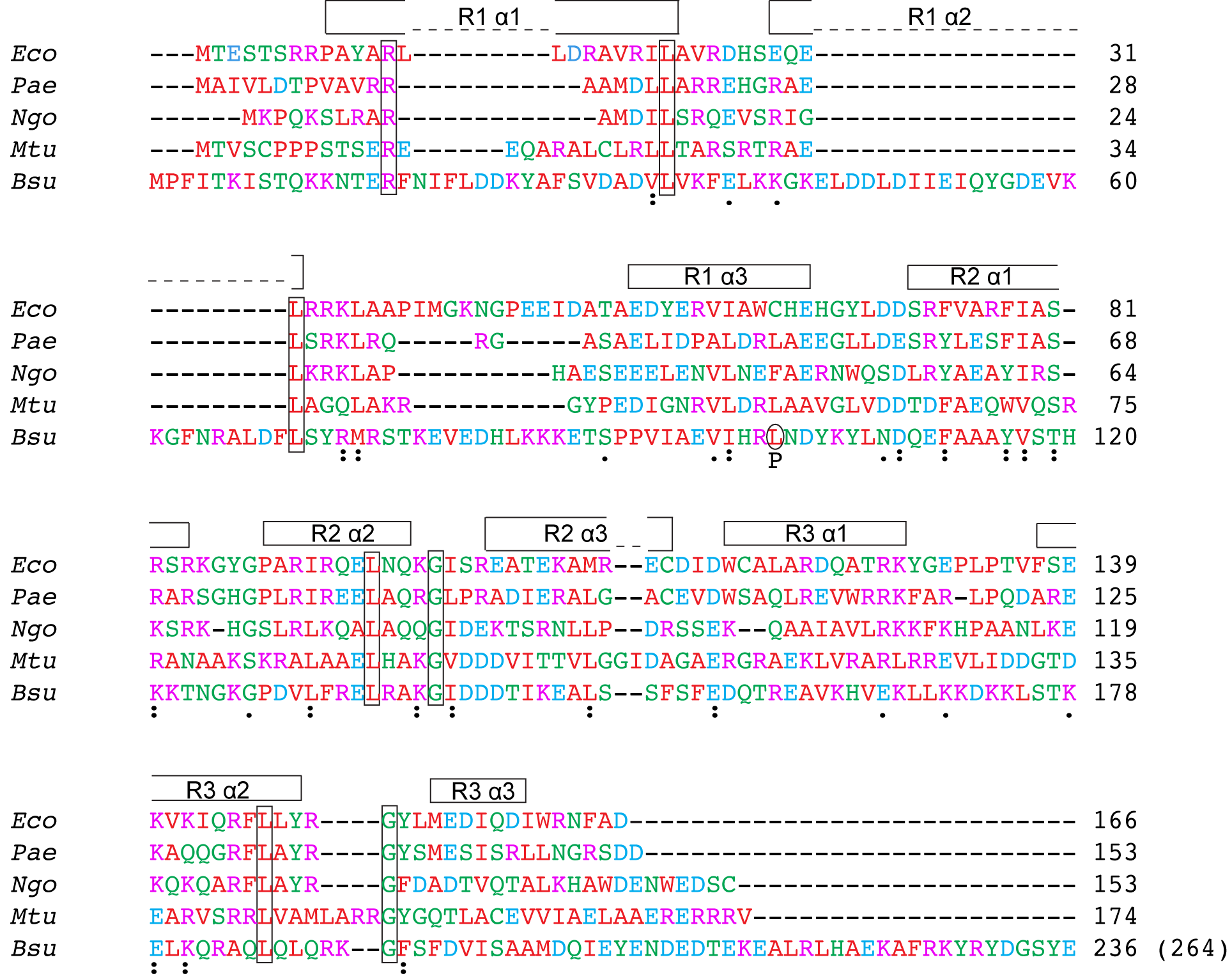

Supplement: Figure S1 — Multiple sequence alignment of representative RecX homologues. Dashes represent gaps introduced to optimize sequence alignment. Abbreviations, accession numbers and Phylum are indicated: Escherichia coli (Eco, A7ZQC76, γ-Proteobacteria), Pseudomonas aeruginosa (Pae, P37860, γ-Proteobacteria), Neisseria gonorrhoeae (Ngo, Q5F7W3, β-Proteobacteria), Mycobacterium tuberculosis (Mtu, P0A5U8, Actinobacteria), and Bacillus subtilis (Bsu, 031575, Firmicutes). RecX sequences were aligned using ClustalW2. The position of the three α-helices for each of the three repeats (R1 to R3), derived from the E. coli RecX X-ray structure, are marked as rectangles above the amino acid sequence. An α-helix is split when there is an insertion that carries potential α-helix breakers. The wild type residue, RecXL101, is encircled, and the residue presented in the RecX342 strain indicated. The color code for the amino acids is the one defined by the ClustalW2 program. The fully conserved residues are framed. Colons and periods, respectively, indicate the strong and weak similar residues. The polypeptide length is indicated, and between parenthesis the full-length. (TIF) [file pgen.1003126.s001.tif]

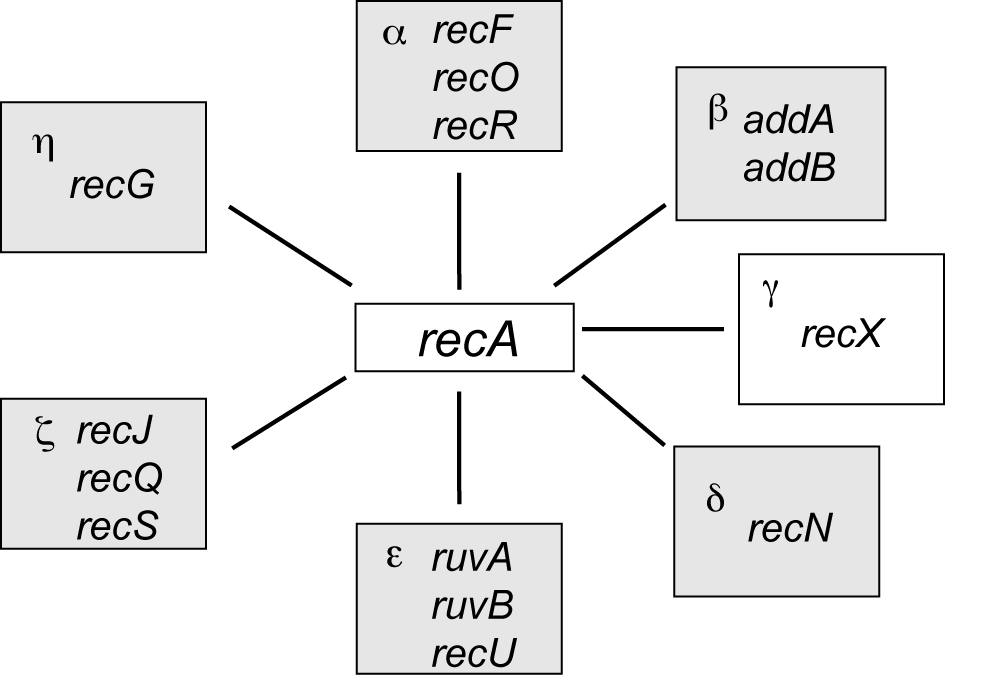

Supplement: Figure S2 — Schematic grouping of B. subtilis RecA-dependent DNA recombinational repair genes into different epistatic groups (α to η). Since a recA mutation is epistatic with any representative mutation of the different epistatic groups, it was placed in the center. The recX (recX342) mutation was placed within the γ epistatic group. (TIF) [file pgen.1003126.s002.tif]

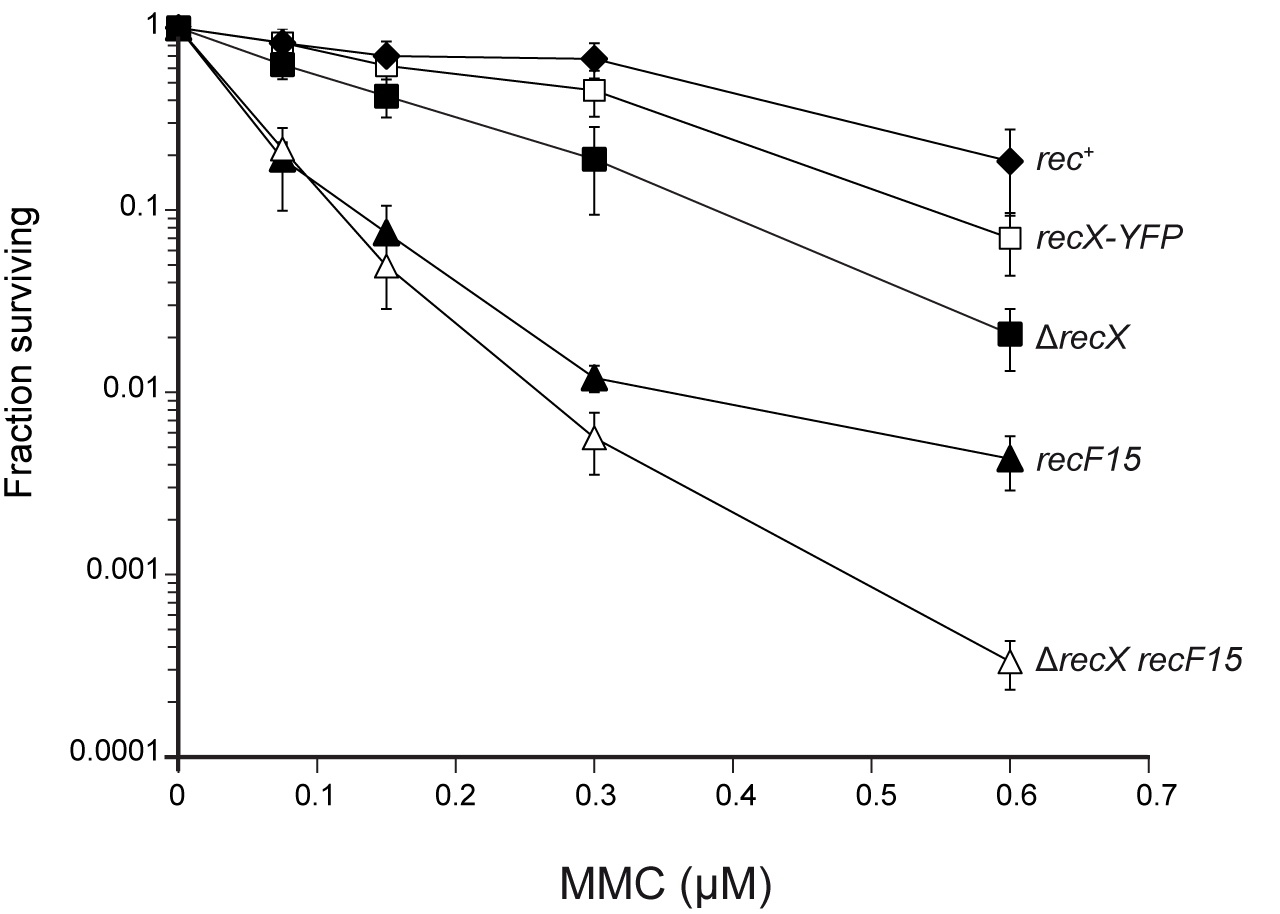

Supplement: Figure S3 — The B. subtilis recX-yfp fusion is fully functional. Cells were grown to OD560 = 0.4 in LB medium and exposed to increasing concentrations of MMC for 30 min. The strains used are indicated by the relevant mutant phenotype. The recX-yfp strain bears a recX-yfp fusion gene. The results are the average of at least four independent experiments and the standard error is indicated. (TIF) [file pgen.1003126.s003.tif]
