# Supplementary material for: RecX Facilitates Homologous Recombination by Modulating RecA Activities
Source: PLoS Genet. 2012 Dec 20;8(12):e1003126. doi: 10.1371/journal.pgen.1003126 (PMC3527212; doi:10.1371/journal.pgen.1003126)
Supplement: Table S1 — Strains used in this study. (DOCX) [file pgen.1003126.s006.docx]

**Table S1.** Strains used in this study.

| Strain | Relevant mutant phenotype | Reference |
| --- | --- | --- |
| BG214 | *trpC*2, *metB*5, *amyE,* *sigB*37, *xre*1, *att*^SPβ^, *att*^ICE^*^Bs^* | Lab. Collection |
| BG119^a^ | + *recH342* renamed as *recX342* | [[1](#_ENREF_1)] |
| BG129^a^ | + *recF15*^b^ | [[1](#_ENREF_1)] |
| BG190^a^ | + Δ*recA* | [[2](#_ENREF_2)] |
| BG439^a^ | + Δ*recO* | [[3](#_ENREF_3)] |
| BG1047^a^ | + Δ*lexA,* Δ*yneAB* | [[4](#_ENREF_4)] |
| BG1065^a^ | + Δ*recX* | This work |
| BG1147^a^ | + Δ*recX,* Δ*recA* | This work |
| BG1137^a^ | + Δ*recX,* Δ*recO* | This work |
| BG1053^a^ | + Δ*recX, recF15* | This work |
| PY79 | Prototroph, wt | Lab. collection |
| CDS19^c^ | + *recX-yfp* | This work |
| CDS20^c^ | + *recX-yfp*, *lacI-cfp,* OH-endonuclease | This work |
| CDS21^c^ | + *cfp*-*recA, recX-yfp* | This work |
| DK37^c^ | + *gfp*-*recA* | [[5](#_ENREF_5)] |
| AKR06^c^ | + *gfp*-*recA*, Δ*recX* | This work |
| SB19 | Prototroph, wt | Lab. collection |

^a^The strains are isogenic with BG214. ^b^Deletion of the *recF* gene affects the expression of downstream essential genes (*gyrA* and *gyrB*) and cells proliferation. The *recF*15 allele, with a single amino acid substitution, does not show a growth defect. The *recF*15 allele shows no residual activity in RR or GR [[6](#_ENREF_6)] and it was used in this study. ^c^The strains are isogenic with PY79.

References

1. Alonso JC, Tailor RH, Luder G (1988) Characterization of recombination-deficient mutants of *Bacillus subtilis*. J Bacteriol 170: 3001-3007.

2. Ceglowski P, Luder G, Alonso JC (1990) Genetic analysis of *recE* activities in *Bacillus subtilis*. Mol Gen Genet 222: 441-445.

3. Carrasco B, Cozar MC, Lurz R, Alonso JC, Ayora S (2004) Genetic recombination in *Bacillus subtilis* 168: Contribution of Holliday junction-processing functions in chromosome segregation. J Bacteriology 186: 5557-5566.

4. Cardenas PP, Carrasco B, Sanchez H, Deikus G, Bechhofer DH, et al. (2009) *Bacillus subtilis* polynucleotide phosphorylase 3'-to-5' DNase activity is involved in DNA repair. Nucleic Acids Res 37: 4157-4169.

5. Kidane D, Graumann PL (2005) Dynamic formation of RecA filaments at DNA double strand break repair centers in live cells. J Cell Biol 170: 357-366.

6. Alonso JC, Stiege AC (1991) Molecular analysis of the *Bacillus subtilis recF* function. Mol Gen Genet 228: 393-400.
