# Supplementary material for: RecX Facilitates Homologous Recombination by Modulating RecA Activities
Source: PLoS Genet. 2012 Dec 20;8(12):e1003126. doi: 10.1371/journal.pgen.1003126 (PMC3527212; doi:10.1371/journal.pgen.1003126)
Supplement: Text S1 — Annex 1. The absence of RecX does not increase the spontaneous mutation rate. Annex 2. recP149 mutation maps is the recA gene. (DOCX) [file pgen.1003126.s007.docx]

**RecX stimulates homologous recombination by modulating RecA activities**

Paula P. Cárdenas^1^, Begoña Carrasco^1^, Clarisse Defeu Soufo^2^, Carolina E. César^1^, Katharina Herr^2^, Miriam Kaufenstein^2^, Peter L. Graumann^2^, and Juan C. Alonso^1,^^[[1]](#footnote-1)^*

^1^Centro Nacional de Biotecnología, CSIC, C/Darwin 3, 28049 Madrid, Spain, and ^2^Mikrobiologie, Fakultät für Biologie, Universität Freiburg, Schänzle Straße 1, 79104 Freiburg, Germany.

Annex 1. The absence of RecX does not increase the spontaneous mutation rate.

To determine whether the reduced threshold for SOS response in Δ*recX* could facilitate the emergence of error-prone DNA repair [[reviewed by 1](#_ENREF_1)] and indirectly contribute to sexual isolation (see Introduction) the spontaneous mutation rate (to rifampicin resistance or reversion of the *metB*5 point mutation to *met*^+^ auxotrophy) under conditions of full SOS induction of *rec*^+^ cells (0.6 μM MMC) was measured. The frequency of spontaneous mutations obtained in *rec*^+^, *recX*342 or Δ*recX* cells in either presence or absence of MMC was not significantly different (data not shown), suggesting that the potential contribution of error-prone repair, if any, is not significant.

Annex 2. *recP*149 mutation maps is the *recA* gene

Three qualitatively distinct situations may arise when the frequency of recombinational repair of single and double mutant strain deficient in recombination (*rec*^-^) are analyzed: (i) the survival frequency may be equal to that of the more deficient single-mutant parent (equal epistatic group), (ii) it may be equal to the sum of each of the single-mutant parents (different group, additive effect) or (iii) it may be greater than the sum of each of the single-mutant parents (different group, synergistic effect) [[see 2](#_ENREF_2)]. However, these analyses are not valid for *recA* mutants, because homologous recombination is going trough the RecA pathway [[3](#_ENREF_3)]. In other words, the survival frequency of any mutant in concert with a mutation in *recA* must be equal to that of the more deficient single-mutant parent (*recA*). In early nineties the *recH*342 (*recX*342) and *recP*149 mutations were tentative classified together within epistatic group γ [[2](#_ENREF_2)]. Both mutants lacked a direct selection, and the double mutant could not be constructed, because the *recP*149 mutation (mapped in the *purA* - *cysA* region, 11º interval, by PBS1 transduction) was incorrectly mapped (Manfredi et al., to be published elsewhere). Recently, the *recP*149 mutation was mapped within the *recA* gene, at the 150º interval, hence *recP*149 was renamed as *recA*149 (Manfredi et al., to be published elsewhere), and withdrawn from epistatic group γ.

**References**

1. Patel M, Jiang Q, Woodgate R, Cox MM, Goodman MF (2010) A new model for SOS-induced mutagenesis: how RecA protein activates DNA polymerase V. Crit Rev Biochem Mol Biol 45: 171-184.

2. Alonso JC, Stiege AC, Luder G (1993) Genetic recombination in *Bacillus subtilis* 168: effect of *recN*, *recF*, *recH* and *addAB* mutations on DNA repair and recombination. Mol Gen Genet 239: 129-136.

3. Ayora S, Carrasco B, Cardenas PP, Cesar CE, Canas C, et al. (2011) Double-strand break repair in bacteria: a view from *Bacillus subtilis*. FEMS Microbiol Rev 35: 1055-1081.

1. *Email address: jcalonso@cnb.csic.es (J.C.Alonso) [↑](#footnote-ref-1)
